# Assessing Paleo-Biodiversity Using Low Proxy Influx

**DOI:** 10.1371/journal.pone.0065852

**Published:** 2013-06-11

**Authors:** Olivier Blarquez, Walter Finsinger, Christopher Carcaillet

**Affiliations:** 1 Centre for Bio-Archaeology and Ecology, Université Montpellier 2, Centre National de la Recherche Scientifique, Montpellier, France; 2 Paleoenvironments and Chronoecology, École Pratique des Hautes Études, Montpellier, France; 3 Centre for Forest Research, Université du Québec à Montréal, Montréal, Québec, Canada; The Pennsylvania State University, United States of America

## Abstract

We developed an algorithm to improve richness assessment based on paleoecological series, considering sample features such as their temporal resolutions or their volumes. Our new method can be applied to both high- and low-count size proxies, i.e. pollen and plant macroremain records, respectively. While pollen generally abounds in sediments, plant macroremains are generally rare, thus leading to difficulties to compute paleo-biodiversity indices. Our approach uses resampled macroremain influxes that enable the computation of the rarefaction index for the low influx records. The raw counts are resampled to a constant resolution and sample volume by interpolating initial sample ages at a constant time interval using the age∼depth model. Then, the contribution of initial counts and volume to each interpolated sample is determined by calculating a proportion matrix that is in turn used to obtain regularly spaced time series of pollen and macroremain influx. We applied this algorithm to sedimentary data from a subalpine lake situated in the European Alps. The reconstructed total floristic richness at the study site increased gradually when both pollen and macroremain records indicated a decrease in relative abundances of shrubs and an increase in trees from 11,000 to 7,000 cal BP. This points to an ecosystem change that favored trees against shrubs, whereas herb abundance remained stable. Since 6,000 cal BP, local richness decreased based on plant macroremains, while pollen-based richness was stable. The reconstructed richness and evenness are interrelated confirming the difficulty to distinguish these two aspects for the studies in paleo-biodiversity. The present study shows that low-influx bio-proxy records (here macroremains) can be used to reconstruct stand diversity and address ecological issues. These developments on macroremain and pollen records may contribute to bridge the gap between paleoecology and biodiversity studies.

## Introduction

Although biodiversity-loss rates are actively investigated to determine their relationship with habitat fragmentation, climatic and social changes (e.g. [1]), there is also a need to assess current rates against longer-term loss rates that occurred in response to past environmental change. Such assessments could be valuable for highlighting the influence of ecological stress and disturbance on biodiversity [2].

In purely theoretical terms, the richness (*S*) of communities at any period of time can be defined as the entire number of species living in the particular area of interest. Among several indices of species richness, the rarefaction method [3]–[5] is often used because it is effective for standardizing the species richness of assemblages of different sizes to a common number of individuals or samples, i.e., the ‘expected taxonomic richness, *E*(*Tn*)’. This standardization is necessary due to the non-linear relationship between richness and the number of individuals recorded. Since Sanders’ original formulation [4] and the subsequent correction by Heck et al. [3], this method has been often used for paleoecological issues to estimate taxonomic richness from (sub)fossil records, where sample size is not well controlled [5], [6]. Examples include terrestrial plants (based on pollen e.g. [7], [8], [9]), algae (based on diatoms e.g. [10]), insects (from chironomids e.g. [11]) or testate amoebae [12].

However, there is increasing awareness of the application of rarefaction to fossil records [13], [14]. Among the assumptions made when estimating past richness using the number of taxa (rarefied or not) in sedimentary samples, the following factors have been critically addressed: (i) count sizes [15]; (ii) the effect of evenness [14], [16]; (iii) differential productivity and dispersal of taxa [14]; (iv) lack of taxonomic precision [6]; (v) the spatial scale for which the sub-fossilized remains are representative of the population actually producing them [17]; and (vi) the temporal resolution of samples [18]. An additional restriction on estimating past taxonomic richness from paleoecological records is that only a fraction of species living in a particular area can become archived.

Changes in floristic richness are commonly inferred from pollen records because of their efficient dispersal and their abundance in sediments [6]. In contrast, despite the growing number of macroremain records [20] that are suitable for reconstructing vegetation-stand dynamics [19], [20] and biomass [21], taxonomic richness has been rarely estimated based on plant macroremains mainly because of mathematical difficulties associated with manipulating the low counts that are frequently recorded [22]. However, tandem studies of pollen and macrofossils confirm the different spatial smoothing and taxonomic resolution of the two types of data, with macroremains providing records of vegetation composition and species ranges with higher spatial details and, for some taxa, higher taxonomic resolution [23]–[25].

Here we describe a new method to estimate and compare the taxonomic richness and evenness of vegetation based on pollen and plant-macrofossil records. Van der Knaap [16] proposed the use of pollen influxes to assess palynological richness and to cope with differential evenness of pollen assemblages. In addition to van der Knaap [16], we introduced the use of interpolated influx that may be regarded as a way to deal with potential pitfalls associated with the species-time-area type relationships, and assumed that rarefaction would be more valuable when used to handle sampling-effort discrepancies within equally sized samples both in terms of volume and time [26]. Finally, we specifically address some issues that are of concern for estimating richness from paleorecords in general, i.e., different temporal resolutions, sample volumes and low-count sizes.

## Materials and Methods

### Species, Volume, Count Size, and Time Relationships

Usually sediments are subsampled on cores collecting samples of equal volume (cm^3^) and thickness (cm on depth) at set intervals resulting in a series of snapshots each of which gives an integrated picture for different periods of time. Depending on the sediment-accumulation rate (yr.cm^−1^), these snapshots represent anything between 1 and >100 years. Furthermore, count sizes tend not to be standardized within a sequence and it sometimes happens that sample volume varies, e.g. for the analysis of plant macroremains. The potential bias for richness estimates related to the variations in sample volume, temporal sample resolution, and count sizes is, in principle, equivalent to “species-time-area relationships” in modern vegetation relevés [18], [27], [28]. In fact, differences in taxonomic richness and evenness may occur between samples of the same sedimentary sequence because higher taxa numbers can be expected in samples of larger volumes, in samples that accumulated over a longer time span, and when count sizes are larger.

Therefore, before estimating richness, counts were resampled to a constant sample resolution (yr.sample^−1^) and sample volume (cm^3^). To do this, we first interpolated the initial sample depths at a constant time interval (*w*) by interpolating between dated top and base of each sample using the age∼depth model. Subsequently, we determined the contribution of initial counts and volumes to each interpolated sample; this step involved the calculation of a proportion matrix. Finally, the interpolated counts were transformed to concentrations (numbers.cm^−3^) that were in turn converted to influxes (numbers.cm^−2^.yr^−1^) by multiplying concentrations by sediment accumulation rate (cm.yr^−1^) for each sample, resulting in a regularly spaced time series of pollen or macroremains. This procedure uses the pretreatment method of charcoal records for reconstruction of fires as proposed by Long et al. [29] and was adapted for the pollen and macroremain records by modifying the interpolation procedure from the freely available CharAnalysis software {https://sites.google.com/site/charanalysis/Higuera, 2009 #308}.

### Individual Based Rarefaction Method

To estimate taxonomic richness [*E*(*T_n_*)] based on rarefaction analysis, we used Heck et al. [3] equation, which was first applied in the context of paleoecology by Birks & Line [6]:
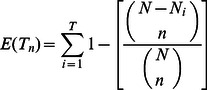
(1)where E(Tn) is the rarefied taxonomic richness, N is the influx sum of particles in a sample, Ni is the influx of particles for the ith taxon, and n the minimum influx along a sedimentary record. This method is particularly suitable for proxies characterized by large total numbers of particles and taxa per sample (e.g. pollen records) that allow high-influx sizes. In contrast, the total number of taxa and particles per sample of low-influx proxies (e.g. macroremains) are often equal to zero. In situations where n = 0, the rarefaction cannot be calculated. To avoid that within a resampled record n = 0, we used the procedure described in the previous paragraph (Species, volume, count size, and time relationships) with increasing resampling time intervals w and selected the minimal w for which all resampled samples displayed influx sums larger than zero. This procedure was not necessary for the pollen records where the minimum influx sums were always >>0, and the records could be resampled using a w equal to the median time resolution.

Rarefaction is sensitive to sample size (e.g. [30]). Furthermore, calculating rarefaction on low influxes such as those usually observed for macroremain records (usually <1 particle.cm^−2^.yr^−1^) would result in richness estimates difficult to interpret. To circumvent this problem, we used a simple conversion procedure of the interpolated taxa matrix to obtain a new converted matrix in which the initial minimum influx sum *n* was converted to *n’*. This procedure was replicated 500 times with increasing *n’* (*n’* = 1,2…500) and, rarefaction was calculated on each of those matrices.

To analyze the long-term richness patterns, we rescaled the *E*(*T_n_*) time series using a simple minimax transformation by subtracting the minimum *E*(*T_n_*) value and dividing by the range of *E*(*T_n_*) values. To assess the significance of *E*(*T_n_*) changes through time, median *E*(*T_n_*) values for adjacent 1000 years’ time windows were compared using a non-parametric Kruskal Wallis ANOVA. When a significant difference was revealed, post-hoc least-significant difference tests were realized to assess the significance between pairs of time periods.

### Evenness

Biodiversity estimates are a combination of the taxa number and the species evenness [31], [32]. Therefore, we also estimated the evenness of fossil assemblages using Hurlbert’s probability of interspecific encounter (Δ1, [33]) such as:
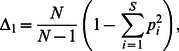
(3)were *p_i_* is the proportion of the *i* ^th^ taxon and *S* the total number of taxa in the sample. Finally, we considered important to distinguish between functional groups in the fossil assemblages, i.e. between tree, shrubs and herbs taxa. This allowed us to highlight the main temporal trends of plant community composition.

The pollen data from Lac de Fully can be accessed from the European Pollen Database (http://www.europeanpollendatabase.net/), the macroremain data is available at http://doi.pangaea.de/10.1594/PANGAEA.807921. The statistical codes and functions were developed under Matlab language (tested using Matlab R2011b) and are freely available at http://blarquez.com/page/Codes/.

### The Test Dataset

In order to test the new algorithm, we used the macroremain and pollen records from Lac de Fully (2135 m a.s.l), a small subalpine lake located on a south-facing plateau in the Valais (inner Swiss Alps). A full description of the site, sediment sampling, the age∼depth model, sample processing, and identification of plant macroremains (e.g. needles, leaves, seeds, bud scales) and pollen is presented in Finsinger and Tinner [34]. The pollen and macroremain records from Lac de Fully cover the past ∼11,000 years. In total, 84 samples were analyzed for macroremains ([min-max] resolution: [24–351] years.sample^−1^) and 62 samples were analyzed for pollen ([50–379] years.sample^−1^) [34]. All ages are expressed in calibrated years before the present (hereafter “cal BP”; by convention the “present” year is AD 1950). In this study we chose to focus solely on the period between 11,000 and 3,000 cal BP, because only four macroremains samples were available for the past ∼3,000 years.

## Results

**Figure 1:**
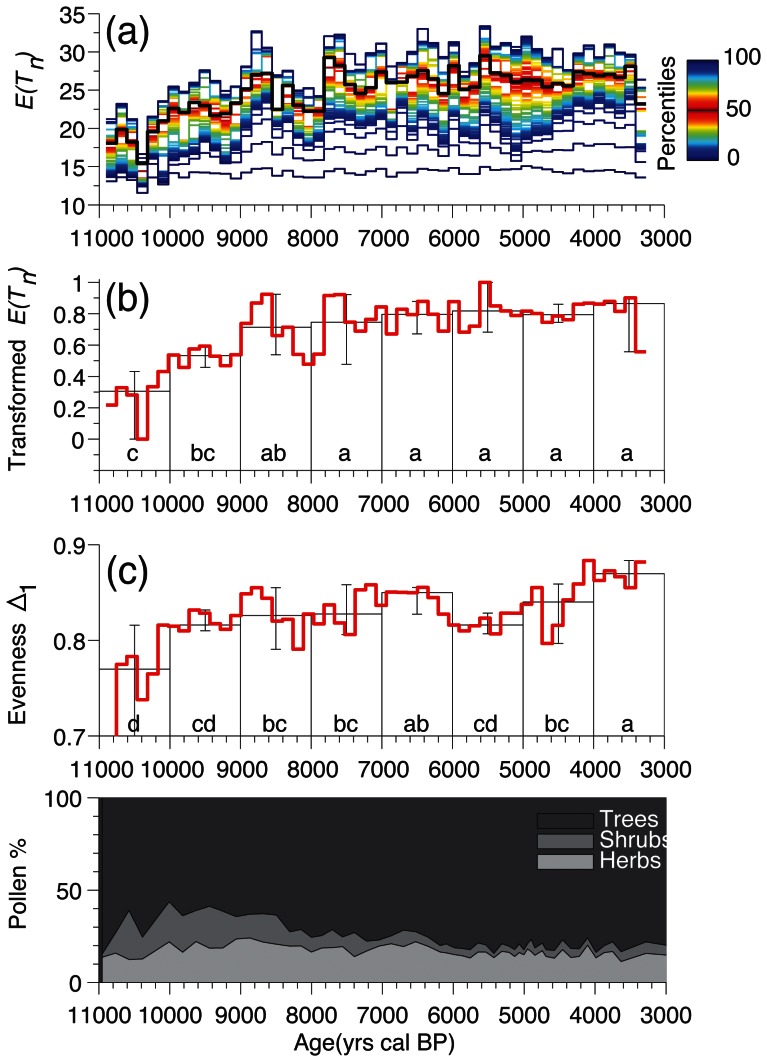
Pollen richness *E*(*Tn*) inferred based on the rarefied and resampled pollen record from Lac de Fully. (**a**) *E*(*Tn*) of all 500 simulations with median as black continuous line and percentiles (see legend) as coloured lines; (**b**) Minimax rescaled median *E*(*Tn*). (**c**) Evenness (Δ_1_) based on the probability of interspecific encounter. The bars in (**b**) and (**c**) indicate the median *E*(*Tn*) and Δ_1 _for 1000-years windows, vertical whiskers indicate the full range of *E*(*Tn*) and Δ_1 _in each time period; letters (a-d) illustrate significant differences from the post hoc least significant difference tests (two different letters indicate a significant difference at the *p = *0.05 level). (**d**) Cumulative pollen percentages of trees, shrubs and herbs.

**Figure 2:**
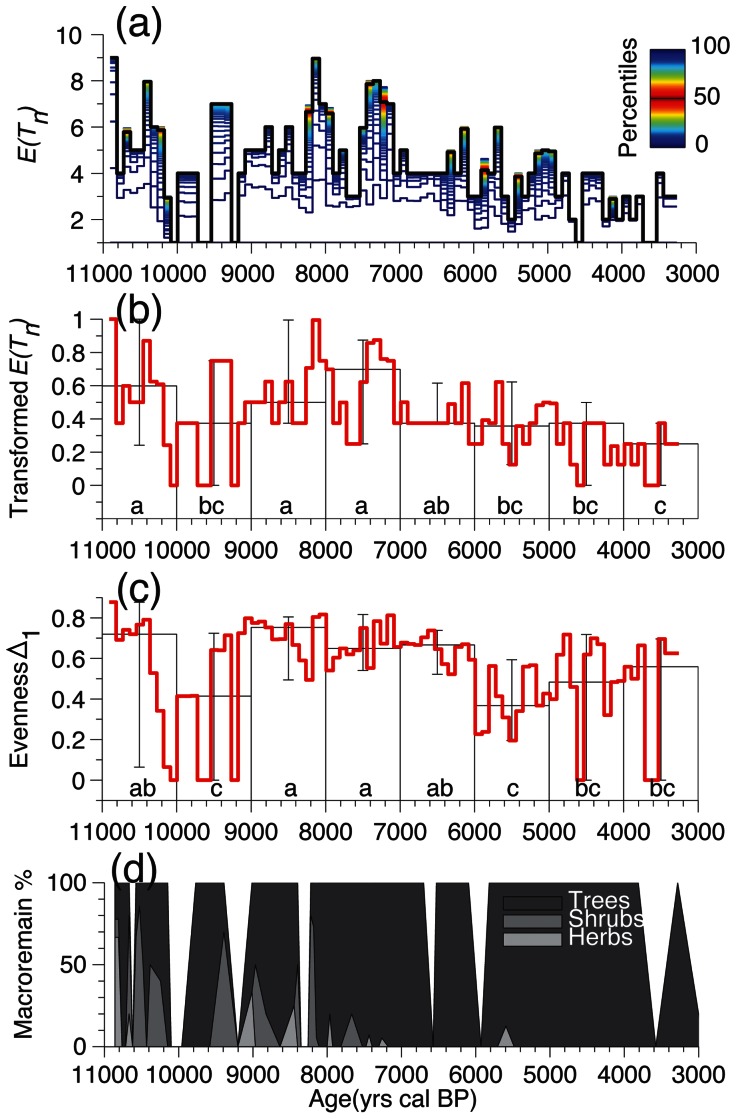
Macroremain richness *E*(*Tn*) inferred based on the rarefied and resampled pollen record from Lac de Fully. (**a**) *E*(*Tn*) of all 500 simulations with median as black continuous line and percentiles (see legend) as coloured lines. (**b**) Minimax rescaled median *E*(*Tn*). (**c**) Evenness (Δ_1_) based on the probability of interspecific encounter. The bars in (**b**) and (**c**) indicate the median *E*(*Tn*) and Δ_1 _for 1000-years windows, vertical whiskers indicate the full range of *E*(*Tn*) and Δ_1 _in each time period; letters (a-c) illustrate significant differences from the post hoc least significant difference tests (two different letters indicate a significant difference at the *p = *0.05 level). (**d**) Cumulative macroremain percentages of trees, shrubs and herbs.

The minimal interpolation time windows *(w)*, that satisfied the condition that any resampled macroremain influx value must be greater than zero, was 91 years for the macroremain record. For the pollen record we interpolated the influxes using the median resolution w = 147 years. The *E(T_n_)* values from the pollen record (Fig. 1a) depict a larger dispersion compared to the macroremain record, which showed more conservative rarefaction reconstructions (Fig. 2a). Estimated richness for the pollen record range from c. 12 to 34 taxa during the 11,000–3,000 cal BP period, and was lower for the macroremain records which showed a maximum *E(T_n_)* of c. 9 taxa (Fig. 2a). The pollen records displayed an evenness that ranged between 0.7 and 0.9 which is generally higher compared to macroremain Δ_1_ that peaked at 0.8 at maximum (Fig. 1c, 2c).

### Pollen Richness and Evenness

Pollen richness (Transformed *E*(*Tn*), Fig. 1b) showed lowest values recorded between 11,000 and 10,000 cal BP (*p*<0.05). After this, *E*(*Tn*) gradually increased up to 8,000–7,000 cal BP; this was accompanied by a gradual decrease in the percentage of shrubs in the pollen assemblages (Fig. 1d; mainly *Corylus*, data shown in Finsinger and Tinner, [34]). Then richness remained stable until the end of the record (p>0.05). *E*(*Tn*) scores were maximal around 5,500 cal BP, when the percentages of tree pollen was high, mainly *Pinus cembra* and *Abies alba* [34]. The evenness of pollen assemblages (Fig. 1c) followed the same general trend as *E*(*Tn*) values (Fig. 1b). The main Δ_1_ pattern is represented by a millennial monotonic increase in Δ_1_ during the early Holocene and a maximum between 4,000 and 3,000 cal BP that is significantly different from the other periods (except the 7,000–6,000 cal BP period, *p*<0.05, Fig. 1c).

### Plant Macroremain Richness and Evenness

The transformed *E*(*Tn*) values for plant macroremains (Fig. 2b) gradually increased from 9,000 to 7,200 cal BP in parallel with a decrease in the percentages of shrub and herb macroremains (mainly *Dryas octopetala* and *Juniperus communis* subsp. *nana*, data not shown, Fig. 2d) and an increase in tree taxa macroremains (mainly *Larix decidua* and *Pinus cembra*, data not shown), indicating either an increase in the altitude of the treeline or increasing woodland density at Lac de Fully [34]. The onset of the Holocene (11,000 to 10,000 cal BP) showed high and gradually decreasing transformed *E*(*Tn*) values that are equivalent to the values recorded between 9,000–6,000 cal BP (*p*>0.05, Fig. 2b). Finally, *E*(*Tn*) decreased between 7,200 and 3,000 cal BP, reaching values close to those from 10,000–9,000 cal BP at about 4,000–3,200 cal BP. The evenness of the macroremain record displayed the same temporal trend compared to richness; however we could report a greater stability in Δ_1_ during the 9,000–6,000 period compared to *E*(*Tn*) (Fig. 2c). Likewise the decrease in Δ_1 _from 7,000 to 3,000 cal BP is less apparent but characterized by a higher variability of Δ_1_ scores (Fig. 2c).

## Discussion

We developed an algorithm allowing the comparison of past trajectories of plant diversity, which is expressed in terms of richness and evenness, as inferred from pollen and macroremain records. We applied our original method to sedimentary data from a subalpine lake located near the Alpine treeline. Solving the problem of low-influxes, when assessing biodiversity, is not trivial. This process involved several steps including a pretreatment of the records and a replication of the rarefaction calculation using multiple deterministic parameters. These problems are important, particularly for plant macroremains [22], certainly for animal remains such as insects or land snails [11], [35] and, testate amoebe in lake sediment in which they are few compared to peat in which they abound [12].

Low-influx sum records pose difficulties for quantitative reconstructions of environmental variables and when investigating biotic responses to environmental changes, because the relative abundances of taxa cannot be accurately estimated [36]. Clearly, low-influx sums also adversely affect the ability to reconstruct changes in taxonomic richness or evenness. The absence or the low concentration of macroremains of certain taxa in most archives used by paleoecologists (e.g. the central part of large lake) cannot be always attributed to their absence from the area surrounding the study site [20]. Of course, if the absence of macroremains is purely due to taphonomic reasons (poor preservation, excessive distance from lake shore, etc.), then it would be advisable to exclude such samples from the record before estimating *E*(*Tn*) [11].

Macroremain records are largely dominated by woody taxa (trees and shrubs) because their plant tissues are better preserved in sediments than plant tissues of non-woody plants, which are mostly composed of cellulose [20], and because they are dispersed over longer distances. The attempts to calibrate the abundance of macroremains in terms of woody biomass demonstrate that such estimates may be affected by large uncertainty [21]. Nevertheless, phases characterized by low concentration (or absence) of macroremains near the treeline can indicate treeline shifts in response to climate changes [37], [38]. In such cases conserving low-influx samples for rarefaction analysis is advisable since they can have an ecological meaning in such environments.

Consequently, to solve the problem of low-influxes, our approach involving the calculation of influxes at regular intervals while maintaining the dependency through the original samples (based on count and volume interpolation, [29]) allows the use of rarefaction on the macroremain record. Our results indicate that, at the study site, floristic richness increased gradually between 10,000 and 7,000 cal BP. Pollen and macroremain records indicate a decrease in relative abundances of shrubs and an increase in trees, pointing to a treeline shift, likely in response to the early Holocene temperature increase (e.g. [25], [39]). Shrub cover can constitute an important component of woody biomass in the subalpine belt [40], [41]. In such shrub-dominated ecosystems, floristic richness is lower than in adjacent conifer-forest stands [42] because shrub cover reduces tree productivity and recruitment [43]. The observed increasing floristic richness during the early Holocene at Lac de Fully may be related to the decrease in shrub cover that was associated with increasing tree cover. This gradual afforestation of the subalpine belt at Lac de Fully during the early Holocene, which contrasts with the more rapid afforestation at other sites in the Central Alps, can be interpreted as reflecting the effect of local climatic conditions. These may have delayed the expansion of closed stands of coniferous trees in the catchment of Lac de Fully until *c*. 8,200 cal. BP, when the climate shifted to more humid and less continental conditions [38].

Diversity indices such as *E*(*T_n_*) include both taxonomic richness and evenness [44]. For both pollen and macroremain records evenness and richness follow similar trends (Fig. 1b *vs* 1c; 2b *vs* 2c), thus confirming the difficulty in distinguishing these two aspects of diversity [6], [16], [32]. However, from our data, we cannot rule out that ecosystems have a higher richness when all pollen and macroremain types co-dominate.

### Conclusion

The present study shows that low-influx paleoecological proxy records (here macroremain records) can be used to reconstruct local scale woody diversity. This suggests the need for further research, including the development of diversity indices and their application for plant communities as described herein for the rarefaction index. Such studies could provide a unique opportunity to analyze precisely the spatial and temporal dynamics of biodiversity across different ecological gradients, including productivity [45] and elevation [24], [46]. Such reconstructions may also facilitate discussions about the long-term impacts of stresses (land-use, climate) or disturbances (e.g. fire, insect outbreaks, extreme climatic events) on the biodiversity of ecosystems and provide reference data for use in predicting relationships between environmental forcing, ecosystem functioning and biodiversity.
